# Editorial Notes

**Published:** 1911-03

**Authors:** 


### Editorial Notes.

**Sanatoria and Tuberculin Dispensaries.**

For the following note we are indebted to Dr. Rowland Thurnam, of Nordrach-on-Mendip :—

*Vaccine Therapy.*—The application of vaccine therapy to so widespread a disease as tuberculosis is of the utmost importance.

All vaccine therapy is difficult, and of all the diseases perhaps tuberculosis presents the greatest difficulty in treatment. The idiosyncrasies of the patients, the necessity for their intelligent and loyal co-operation with the doctor, even the numerous varieties of tuberculin available, all tend to make the treatment a complicated matter. Those of us who have given tuberculin a fair trial are convinced of its value, especially when given in progressive doses with the object of securing that immunity which large doses give. But all will agree that this vigorous employment of so powerful a remedy is not unattended by risk, unless the most strict vigilance be maintained over the patient's temperature and daily conduct.

It is natural that those who advocate a new method of treatment should refrain from alarming their hearers by enumerating the dangers and difficulties ; but it is by mistakes and failures that we learn, and excessive optimism on the part of pioneers is apt to be followed by disappointment and incredulity. This has happened before in the history of tuberculin.

The attention of the public is just now being strongly directed to the subject of tuberculosis. Lectures and exhibitions are being held all over the country ; consumption is recognised both as a danger to the community and as a preventable and curable disease. Municipalities are now willing to supply the money necessary to cope adequately with the common enemy. It is very important at this juncture that the medical advisers of those bodies should not be so swayed by ideas of economy as to assure the ratepayers that the provision of tuberculin dispensaries will be all-sufficient, and that the cost of cure will not exceed one pound a head ! Dispensaries are needed.

Dispensaries must form a valuable part of the proper equipment of a town against tuberculosis ; but the provision of a dispensary does not do away with the need of a sanatorium.

In pre - tuberculin days a sufficiently long course of sanatorium treatment was almost impracticable for the poor, but it is one thing to take a possible bread-winner from his work and keep him three or four months at a Sanatorium, and quite another thing, both for him and the ratepayers, to give him a sorely-needed rest for a short period, feed him up, start him on a suitable kind of tuberculin (to be continued while attending a dispensary), and let him learn by demonstration and the experience of fellow sufferers, the importance of those hygienic details which characterise sanatorium life. Money would be well spent in that way ; for without such a preliminary education, not only may it be difficult for the doctor to gauge the condition and capabilities of his patients, but the efforts he makes on their behalf may be nullified by their own ignorance and inattention.

One must also bear in mind the fact that the efficacy of a vaccine treatment depends on the power of the patient to respond. We know that an underfed and overworked patient cannot resist disease ; in other words, he has not the wherewithal to manufacture the necessary antibodies which should result from the administration of the vaccine. When tuberculin is being administered Nature strains herself to manufacture antibodies, and may overstrain herself : unless some help be given to the system in the early stages of phthisis, especially rest and extra food, the response to tuberculin is likely to be slow and disappointing. I was much struck by the strained and weary expression of the many patients I saw attending the dispensaries I have visited.

No doubt a few cases, diagnosed in an early stage (*e.g.* contacts), could be treated without the sanatorium ; but I would strongly urge the importance of providing municipal sanatoria as well as dispensaries where patients could spend the first period of their treatment, and to which others who were not making satisfactory progress under dispensary treatment could return if necessary.

